# Frontiers of Autoantibodies in Autoimmune Disorders: Crosstalk Between Tfh/Tfr and Regulatory B Cells

**DOI:** 10.3389/fimmu.2021.641013

**Published:** 2021-03-26

**Authors:** Tingting Ding, Rui Su, Ruihe Wu, Hongwei Xue, Yanyan Wang, Ronghui Su, Chong Gao, Xiaofeng Li, Caihong Wang

**Affiliations:** ^1^Department of Rheumatology, The Second Hospital of Shanxi Medical University, Taiyuan, China; ^2^Pathology, Joint Program in Transfusion Medicine, Brigham and Women’s Hospital/Children’s Hospital and Harvard Medical School, Boston, MA, United States

**Keywords:** B regulatory cells, autoantibody, T follicular helper cell, T follicular regulatory cell, germinal center, autoimmune diseases, immunotherapy

## Abstract

Balance of Tfh/Tfr cell is critically important for the maintenance of immune tolerance, as evidenced by the fact that T follicular helper (Tfh) cells are central to the autoantibodies generation through providing necessary help for germinal center (GC) B cells, whereas T follicular regulatory (Tfr) cells significantly inhibit autoimmune inflammation process through restraining Tfh cell responses. However, signals underlying the regulation of Tfh and Tfr cells are largely undefined. Regulatory B cells (Bregs) is a heterogeneous subpopulation of B cells with immunosuppressive function. Considerable advances have been made in their functions to produce anti‐inflammatory cytokines and to regulate Th17, Th1, and Treg cells in autoimmune diseases. The recent identification of their correlations with dysregulated Tfr/Tfh cells and autoantibody production makes Bregs an important checkpoint in GC response. Bregs exert profound impacts on the differentiation, function, and distribution of Tfh and Tfr cells in the immune microenvironment. Thus, unraveling mechanistic information on Tfh-Breg and Tfr-Breg interactions will inspire novel implications for the establishment of homeostasis and prevention of autoantibodies in diverse diseases. This review summarizes the dysregulation of Tfh/Tfr cells in autoimmune diseases with a focus on the emerging role of Bregs in regulating the balance between Tfh and Tfr cells. The previously unsuspected crosstalk between Bregs and Tfh/Tfr cells will be beneficial to understand the cellular mechanisms of autoantibody production and evoke a revolution in immunotherapy for autoimmune diseases.

## Introduction

Autoimmune disorders encompass a heterogeneous group of diseases in which immune tolerance is broken and the self-immune system mistakenly attack autologous tissues, leading to local and/or systemic damage. In the majority of these diseases, self-reactive lymphocytes and pathogenic autoantibodies are pivotal in inflammation-mediated tissue and organ damage.

The generation of autoantibodies is mainly related to lymphoid follicular germinal centers (GCs), which are microenvironment structures where antigen-specific B cell clones through a multistage differentiation process (1, 2). After priming by antigen, naive B cells undergo somatic hypermutation, affinity maturation, and class switch recombination in a T cell-dependent mechanism in the GCs, and further differentiate into antibody-producing plasma cells and memory B cells. Generally, the GC response is regulated and contributes to the production of antibodies specialized for preventing foreign pathogens. Multiple factors including follicular dendritic cells (FDCs) (3, 4), regulatory T cells (5), and microbiota (6) collaborate to regulate this response. Particularly, T follicular helper (Tfh) cells (7) and T follicular regulatory (Tfr) cells (8–10) are central to the production of antibody in GCs. Tfh cells are characterized by CXC chemokine receptor 5 (CXCR5) and lineage-defining transcription factor B-cell lymphoma 6 (Bcl-6) (11–13). These cells differentiate from naive CD4+T cells after priming by antigen-presenting cells and can access B cell follicles in a CXCR5-dependent manner. During the multistep process of GC reactions, Tfh cells emerge as superior helpers, providing proliferation, selection, and survival signals to cognate B cells, indicating their important role in antibody production (14). However, this help from Tfh cells must be restrained to prevent the production of autoantibodies which underlie unwanted reactions to self-antigens. In 2011, three separate studies initially found a specialized GC-located subset of regulatory T cells (Tregs) required for suppression of the GC response in mice and termed them as Tfr cells (8–10). Since then, Tfr cells in the regulation of immunity have received intense attention. Tfr cells constitutively expressed Foxp3 and CXCR5 and have a phenotype similar to both Treg and Tfh cells. A popular model of their function is to restrain the magnitude of the GC reaction by inhibiting Tfh cell proliferation and self-reactive B cell activation (8–10). Clinical studies have investigated Tfr and Tfh cells in multiple autoimmune diseases and have found a decreased frequency of circulating Tfr cells and an increased Tfh frequency as well as an aberrant Tfh/Tfr ratio in rheumatoid arthritis (RA) (15), systemic lupus erythematosus (SLE) (16) and myasthenia gravis (17). Importantly, the Tfh/Tfr ratios is positively correlated with serum anti-double stranded DNA (dsDNA) antibody level in SLE patients (16). Moreover, previous study transferred Tfr cells *in vivo* to demonstrate their function in restricting autoreactive GC formation and reducing autoantibody-producing B cells (18). Thus, dysregulation of Tfh and Tfr cells may contribute to the production of autoantibodies in autoimmune diseases.

In addition to Tfh and Tfr cells, regulatory B cells (Bregs) exhibit potent regulatory function in autoantibody production through complex interactions with multiple lymphocytes involved in the GC response. Bregs represent a heterogeneous population of B cells possessing immunosuppressive functions through different mechanisms, and there is no lineage-specific transcription factor for the identification of these cells (19). Indeed, the well-established functions of B cells in immune responses are antibody production, pro-inflammatory cytokine secretion and antigen presentation. It was not until the 1970s, that the existence of suppressive subsets of B cells was first confirmed in delayed hypersensitivity reactions (20). In 2002, Mizoguchi et al. (21, 22) reported that interleukin 10 (IL-10)-producing B cells can suppress intestinal inflammation progression in mouse models and first described these cells as Bregs. Currently, various Bregs have been identified, such as CD1d^hi^CD5^+^Bregs (23), CD25^hi^FoxP3^hi^Bregs (24) and Tim-1^+^Bregs (25). The potent regulatory functions of various Bregs have been identified in immune-related pathologies, including inflammation, autoimmunity, and transplantation (22, 23, 26). Several research groups have shown the association between numerically and/or functionally aberrant Bregs and autoimmune diseases such as SLE (27), RA (28), and multiple sclerosis (MS) (29).

There is increasing interest in exploring the regulatory mechanisms of Bregs, especially the interaction with multiple targets cells. CD19^+^CD24^hi^CD38^hi^ Bregs function to maintain the Th1/Th2 and Th17/Treg balance *via* IL-10 (28). Moreover, IL-10-producing Bregs inhibit the function of natural killer (NK) cells (30) and plasmacytoid dendritic cells (31). Recently, clinical studies have shown that the dysregulated Tfr and Tfh cells were correlated with impaired Bregs in many autoimmune disorders (32–34). Immunological advance further suggests the regulatory potential of Bregs on Tfr and Tfh cells in the germinal response (26, 33, 35, 36), providing new implications for understanding the production of autoantibodies in autoimmune diseases.

In this review, we briefly outline recent advances in the biology of Bregs and their involvement in autoimmune diseases. In particular, we focus on the interactions between Bregs and Tfh/Tfr cells and how such interactions regulate autoantibody production in autoimmune diseases. Finally, we discuss the therapeutic implications based on Breg-mediated regulation of Tfh/Tfr cells in autoimmune diseases and propose several problems that needed to be solved regarding this therapy. This will give rise to more effective therapies and monitors for autoimmune disorders.

## Phenotypes of Bregs

Currently, Bregs are primarily defined by their immunosuppressive function *in vitro* or *in vivo*, especially their capacity to produce the anti-inflammatory cytokine IL-10. Diverse B cell subsets with suppressive functions have been identified in experimental animal models and human, although the unique phenotypic markers for distinguishing Bregs from effector B cells have not been unified, due to the strong heterogeneity of Bregs. In mice, Mauri et al. (37) showed that CD1d^+^CD21^+^CD23^+^transitional 2-MZ precursor (T2-MZP) B cells, which secrete IL-10 have immunosuppressive activity both *in vitro* or *in vivo*. Studies have also identified CD21^+^CD23^−^CD24^hi^ MZ Bregs (38, 39) and CD1d^hi^CD5^+^ Bregs (40) with the capacity to produce IL-10 in mice. In addition, IL-10-producing CD19^+^CD138^+^ plasmablasts have been found in experimental autoimmune encephalomyelitis (41). A study also indicated that the cluster of differentiation 9 (CD9) is a functional marker of IL-10-expressing Bregs in mice (42). In other studies, T cell immunoglobulin and mucin-domain-containing protein (Tim-1) is an important marker for murine IL-10-producing B cells (25, 43). In the study by Lino et al. (44), LAG-3^+^CD138^hi^ plasma cells mediated immune regulation in mice. Similarly, CD24^hi^CD38^hi^ immature B cells (27), CD24^hi^CD27+ B cells (45, 46), and CD25^hi^CD71^hi^CD73^lo^ Br1 cells (47) have been reported in humans. Recently, human IL-10-producing IgA+ B cells induced by a proliferation-inducing ligand have been shown to inhibit T cell and macrophage responses (48).

Apart from the most investigated IL-10-producing Bregs, great efforts have been made to identify expanding Breg subsets independent of IL-10. For example, Liu et al. (49) found novel CD11b+ Bregs can suppress CD4+ T-cell responses in mice with experimental autoimmune hepatitis. Moreover, CD38+CD1d+IgM+CD147+granzymeB+ B cells (50) and IL-35-producing Bregs (51) have been identified in humans and mice, respectively. It has also been determined that murine PD-L1hi B cells can suppress humoral immunity (33). B cells expressing IgD at a low level have been identified as a novel population of Bregs in humans (52).

Collectively, the phenotypes of Bregs in different species, organs, and disease models are partially overlapping or distinct, which might due to their adaption to special immune environments (19). Additional studies are needed to determine whether the immunomodulatory function of Bregs relies on their phenotype.

## Origin and Induction of Bregs

The inability to identify different Breg cell subsets in complex immune microenvironment makes it difficult to understand the generation and biological characteristics of Bregs more deeply. Up to now, the origin of Bregs remains elusive and controversial. B cells can be divided into two main populations according to their distinct origins, B1 and B2 B cells. B1 B cells develop in the fetal liver and mainly present in the peritoneal cavity. B2 B cells develop in the bone marrow and further differentiate into marginal-zone (MZ) B cells or follicular B cell after several transitional stages. Some researchers proposed that Bregs with various phenotypes rise from a special progenitor and lineage-defining transcription factors determine their immunosuppressive nature. Early studies have suggested the B1 lineage of IL-10 producing Bregs as B1 B cells are the main source of B cell-derived IL-10 (53). Bregs can also originate from B2 B cells. In one study, Gαi2-deficient mice developed a spontaneous colitis due to the absence of splenic T2-MZP and MZ B cells, although follicular B cells were not altered, suggesting the potential origin of Bregs from T2-MZP and MZ B cells (54). Subsequent studies also demonstrated that T2-MZP can convert into Bregs (37, 55, 56). Notably, the identification of CD19^+^CD5^+^CD1d^high^ thymic B cells with suppressive ability suggest the thymic origin of partial Bregs (57). However, there is no lineage-specific markers have been identified in the gene arrays of Bregs (47, 51), which does not support the hypothesis that Bregs arise from a dedicated progenitor. Indeed, growing evidence has demonstrated that multiple B cells at different stages of development can acquire suppressive functions under special microenvironmental stimulations (27, 41, 58). Moreover, Maseda’s group (59) reported that antigen-specific *in vivo* signals initiated genetic and phenotypic alternations in B10 cells and led to the conversion of these cells into antibody-producing plasmablasts. These facts strongly support the hypothesis that any B cell has the potential to convert into Bregs in response to appropriate stimuli and their regulatory capacity may change with the environmental alternations.

A complete understanding of the differentiation of diverse Breg is needed. Currently, great progresses have been made in understanding the induction of Bregs by microenvironmental molecules, especially inflammatory regulators. As extensively summarized elsewhere, B cell receptor (BCR) recognition, CD40, and Toll-like receptors (TLRs), contribute to the induction of IL-10-producing Bregs (60). Pro-inflammatory cytokines such as IL-6, IL-1β (61), IL-21 (62), interferon gamma alpha (IFN-α) (31), and B cell-activating factor (BAFF) (63) are also potent inducers of Bregs. Pro-inflammatory cytokines-mediated induction of Bregs can be explained as a feedback mechanism that suppresses the expansion of pro-inflammatory cells and restores immune homeostasis. However, it appears that not all pro-inflammatory cytokines play a role in the generation of Bregs. For example, the deficiency of IL-17, a pro-inflammatory cytokine, led to an increased number of CD19^+^IL-10^+^Breg in the spleen of a murine model of lupus (64). Of note, IL-35 can induce IL-35-producing Bregs as well (65), suggesting the potential role of anti-inflammatory cytokines in the differentiation of Bregs. Moreover, gut-microbiota-derived signals are important in the development of IL-10-producing Bregs and drive their differentiation by inducing the production of pro-inflammatory cytokines by DCs and macrophages (61). This was corroborated by study showing that the elimination of gut microbiota through antibiotic treatment led to reduced IL-10-expressing Breg frequency in mice compared to the controls (61). Subsequent studies suggested that intestinal microbiota drove B cells into IL-10-producing Bregs *via* TLR2/MyD88/PI3K signaling (66). Furthermore, stimulation from bacteria-derived oligodeoxynucleotides bas been shown to induce the generation of human CD24^hi^CD38^hi^ Breg-like cells *in vitro* (67). Similarly, the DNA of gut microbiota has been shown to mediate the expansion of Bregs in MRL/lpr mice (68). More studies are needed to determine the mechanism of underlying bacterial DNA-mediated induction of IL-10-producing Bregs.

A recent study identified IL-10^+^LAG-3^+^CD138^hi^ regulatory plasma cells in germ-free mice, suggesting that gut microbiota is not indispensable in the generation of all subsets of Bregs (44). The study also found that these regulatory plasma cells were naturally existed in the spleen and bone marrow of naive mice, rather than develop in response to stimulation. They originate from several B cell subsets including B1a, B1b, and B2 cells in a BCR-dependent manner and produce IL-10 after activation by TLR signals (44). Indeed, the existence of naturally occurring CD19^+^CD25^high^CD27^high^CD86^high^CD1d^high^IL-10^high^ Bregs in humans has also been confirmed (69). A complete understanding of these natural Bregs and their relationship with inducible Bregs is needed. In addition, more studies are needed to explore the origin and development of various Bregs, which will provide novel insights into the therapeutic potential of Bregs in autoimmune disorders.

## Suppressive Mechanisms and Effectors of Bregs

The most investigated regulatory mechanism of Bregs centers around their production of IL-10. Mice with IL-10 knocked-out in B cells developed exacerbated arthritis accompanied by increased antibodies and inflammatory Th1 and Th17 cells, suggesting the important role of IL-10 in B cell-mediated immune regulation (70). Consistently, human CD19^+^CD25^high^ Bregs can inhibit the expansion and function of autologous CD4+ T cells, and promote the differentiation and activity of Tregs *via* secretion of IL-10 (71). Moreover, CD1d^hi^CD5^+^Breg-derived-IL-10 inhibits the activation of IL-13^+^ type 2 innate lymphoid cells (ILC2s) and thereby suppressing the inflammation response in contact hypersensitivity (72). In experimental allergic encephalomyelitis (EAE), regulatory plasmablast-derived IL-10 also inhibits the production of IFN-α by DCs, a key cytokine in the expansion of pathogenic T cells (41).

The IL-10-independent regulatory mechanisms of Bregs are primarily mediated by IL-35 (51), transforming growth factor beta (TGF-β) (73), and granzyme B (50). These cytokines act by inducing Treg and inhibiting effector T cell differentiation (50, 51, 73). Moreover, programmed death-ligand 1 high (PD-L1hi) Bregs inhibit the expansion of Tfh cells and effector T cells through programmed cell death protein 1 (PD-1)/PD-L1 signaling (33). CD1d^+^T2-MZP Bregs functioned to regulate the activity of invariant natural killer (iNKT) cells through CD1d–lipid presentation, thereby inhibiting excessive inflammation (74). Notably, specific IgG4 antibodies produced by human CD73^−^CD25^+^CD71^+^ IL-10-producing regulatory B cells also play an important role in the suppression of antigen-specific CD4+ T cell proliferation (47). Other molecules such as glucocorticoid-induced tumor necrosis factor receptor ligand (52, 75), intercellular adhesion molecule 1, and fasciclin 1 also mediate the suppressive function of several populations of Bregs (76). Recently, the importance of microbiota-derived butyrate, a type of short-chain fatty acid (SCFA) in the function of IL-10-producing CD19+CD21hiCD24hiB cells has been identified. Butyrate activates transcriptional marker aryl-hydrocarbon receptor (AhR) in a manner dependent on 5-Hydroxyindole-3-acetic acid (5-HIAA) and therethrough indirectly supports CD19^+^CD21^hi^CD24^hi^Breg cell function (77). Although butyrate supplementation can support suppressive function of Bregs and inhibits arthritis in mouse models, it is unclear which species of the gut microbiota are involved in SCFA-mediated regulation of Bregs. The efficacy of dietary invention with butyrate also remains to be confirmed in RA patients.

Importantly, several transcription factors important for the suppressive functions of Bregs have also been identified. A study from Florian et al. (78) on the transcriptomic meta-analysis of human Bregs identified two critical immune regulatory transcriptional signatures, *GZMB* and *IL10RA*, among 126 differentially expressed genes between Bregs and non-Bregs. Recent research also suggests an important role for transcriptional repressor B lymphocyte-induced maturation protein-1 (Blimp-1) in the function of activated B10 cells. The authors found that Blimp-1 promoted the transcription of IL-10 when accompanied by phosphorylated signal transducer and activator of transcription 3 (STAT3) (79). Another transcription factor interferon regulatory factor 4, serves as a crucial modulator of TLR signaling, promoting IL-10 secretion by plasmablasts in the draining lymph nodes (LNs) (41). Studies have also identified the high expression of AhR in Bregs, which maintains the phenotype of splenic CD19^+^CD21^hi^CD24^hi^Bregs by regulating IL-10 production and by restricting pro-inflammatory gene expression. This is supported by the fact that mice with AhR deletion developed exacerbated arthritis accompanied by reduced IL-10-producing CD19^+^CD21^hi^CD24^hi^Bregs (80). Hypoxia-inducible factor-1α is also a critical transcription factor which regulate IL-10 expression in B cells (81). However, it is unclear whether these transcriptional determinants of Breg immunosuppressive functions are shared by different Breg subsets. To date, Breg-cell-specific transcription factors are largely undefined, and more studies are required in the future.

The potential role of Bregs in regulating Tfh and Tfr cells may also contribute to suppressing inflammation (33). However, this regulation appears more complex and will be discussed below. In summary, diverse mechanisms of Breg function are associated with the complex interactions between Bregs and other immune cells. Further studies are need to explore the transcriptional mechanisms underlying these interactions.

## Involvement of Tfh and Tfr in Autoimmune Diseases

### Tfh and Tfr Control Autoantibody Production

Autoantibodies are serological markers and pathological contributors to many autoimmune diseases. Autoantibodies can lead to the deposition of immune complex in various organs, thereby activating the complement system and/or activating immune cells, resulting in severe inflammatory damage. Moreover, autoantibodies cause direct damage of target tissue *via* antibody-dependent cell-mediated cytotoxicity (82). Generally, high-affinity antibodies generated in the GC during humoral immunity are responsible for providing long-term protection against the multiple pathogens. Upon antigen recognition, mature naïve B access the GC and further interact with T cells to differentiate into antibody-producing plasmocyte (1). A central step of high-affinity antibody maturation is somatic hypermutation (SHM) of BCR genes. However, in addition to enhancing affinity, this process can give rise to self-reactive BCRs at the same time (83), which underlines autoantibodies production. Although multiple checkpoints exist, including central (84) and peripheral tolerance (85), elimination of massive autoreactive B cells, more direct regulations at the GC level are necessary to prevent autoantibody production.

Since the discovery of Tfh and Tfr cells, the immunological mechanism underlying autoantibody generation in autoimmune diseases gradually becomes clear. Tfh cells are specialized CD4+ T lymphocytes required for the initiation of GC response in humoral immune responses. CXCR5, BCL6 and IL-21 are functional markers commonly used to define these cells. Within the follicle, Tfh cells provide signals for GC-B cell survival, affinity maturation, proliferation, and differentiation (86). These signals include cytokines such as IL-4 and IL-21 (14), and cellular interaction through surface molecules including CD40L (14, 87, 88), ICOSL (89) and PD-1 (90). The interaction between Tfh and GC-B cells is not well defined. It has been reported that the frequency and antigen affinity of B cells determine their capacity to receive help from T cells (91). Normally, only high-affinity B cells successfully compete for Tfh help and undergo rounds of selection to differentiate into antibody-producing plasma cells, whereas other B cells without this help may die rapidly. A series of studies have indicated that excessive Tfh cell response leads to the production of autoantibodies (92–94). It is possible that self-reactive B cells can receive help from excessive Tfh cell signals and escape from tolerance, leading to the generation of autoantibodies.

**Figure 1:**
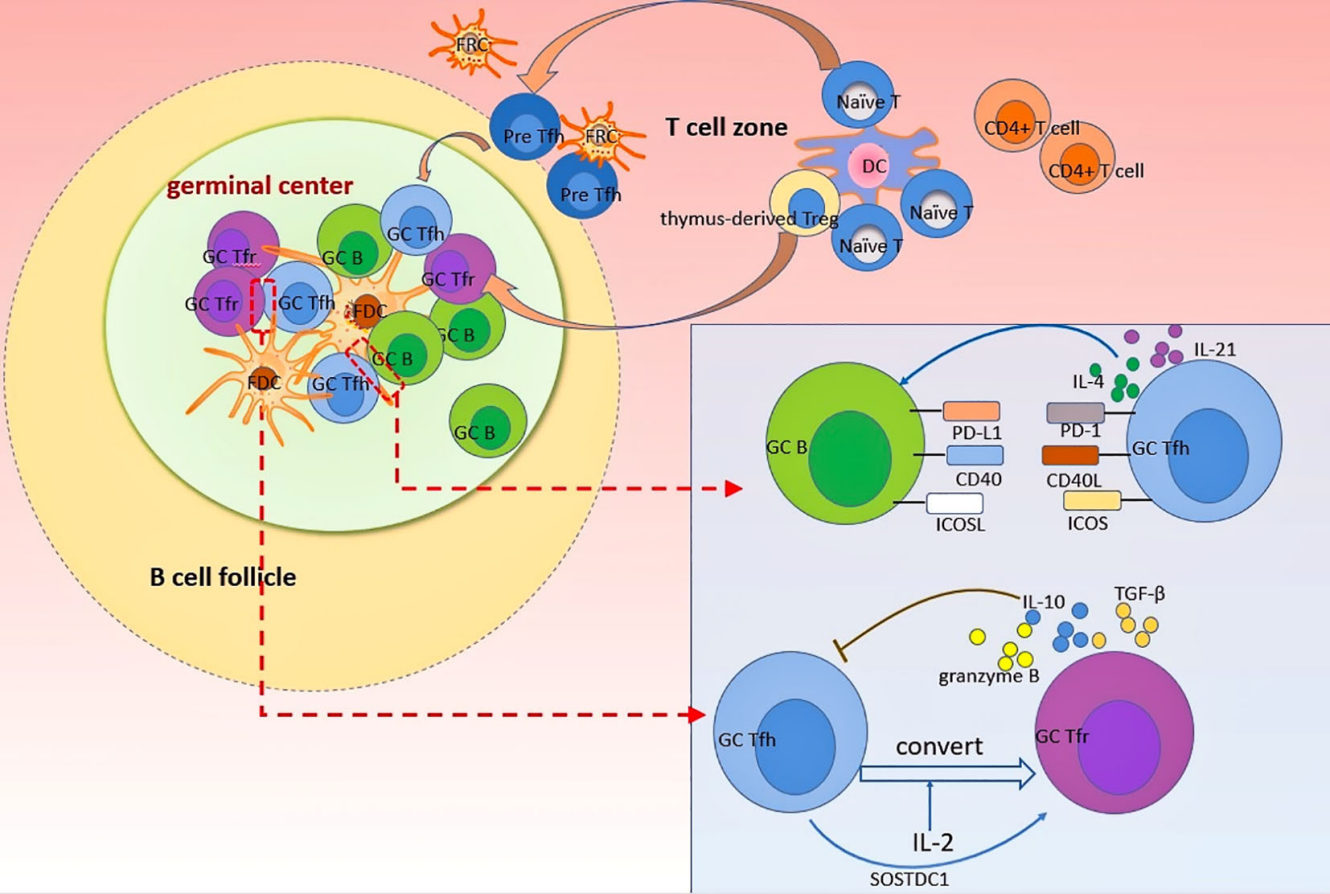
Dynamics of Tfh and Tfr cells in GC response. Naïve T cells and thymus-derived Treg can differentiate into Tfr and Tfh cells, respectively, after the priming by dendritic cells. Differentiated Tfr and Tfh cells gradually migrate into follicles in a CXCR5-dependant manner to exert profound impacts on GC B cells. Follicular stromal cells such as FDC provide an important plat form for various cellular interaction. In GC, Tfh cells support B cells differentiation and antibodies production by providing essential signals to B cells through direct interactions mediated by PD-1, CD40L, and ICOS, as wells as cytokines such as IL-4 and IL-21. By contrast, Tfr cells uniquely inhibit the differentiation and function of Tfh cells through secreting several anti-inflammatory cytokines (including IL-10, TGF-β, granzyme B) to suppress the GC response. The crosstalk between Tfr and Tfh cells is complex. Tfh cells can convert into Tfr cells after stimulation by IL-2. Moreover, SOSTDC1-producing Tfh cells can serve as an inducer of Tfr cells.

Conversely, Tfr cells act as negative regulators of autoantibody generation. Tfr cells are mainly differentiated from thymic forkhead box protein 3 (Foxp3)^+^ Treg precursors and characteristically express Foxp3 and CXCR5. Moreover, other molecules such as Bcl-6 and PD-1 are also expressed in Tfr cells (8). Tfr cells are critically involved in regulating the GC response. On the one hand, Tfr cells inhibit the proliferation and function of Tfh and B cells in GC in a cytotoxic T-lymphocyte-associated protein 4 (CTLA-4)-dependent manner (95, 96). Deletion of CTLA-4 on Tfr cells results in impaired class-switch recombination to IgG1, as well as unrestrained proliferation of Tfh cells (97). On the other hand, Tfr cells act by secreting anti-inflammatory cytokines including IL-10 (98), TGF-β (10, 99), and granzyme B (10) (**Figure 1**). Additionally, Tfr cells seem to impair the B-cell response to Tfh cell stimulation by inhibiting B-cell metabolic pathways, thereby leading to decreased antibody production (96). Importantly, studies of infection models have indicated that Tfr cells eliminate autoreactive B cells in the GC response, suggesting the important role of these cells in maintaining B-cell tolerance (100). This is supported by the fact that ablation of Tfr cells promotes autoantibody production in house dust mite models (101). In another study, mice with impaired GC-Tfr cells due to conditional knockout of nuclear factor of activated T-cells 2 showed increased pathogenic anti-dsDNA and developed lupus-like disease after immunization with chromatin (102). Thus, it could be possible that abnormalities in Tfr cells numbers and functions lead to impaired negative selection of autoreactive B cell and enhanced Tfh activity, which ultimately promotes autoantibodies production in autoimmune diseases.

Given the opposing roles of Tfh and Tfr cells, a balance of them is indispensable for fine tuning the GC response. Multiple factors such as microRNAs (103), gut microbiota (104), and cytokines [especially IL-10 (26), IL-2 (100, 105) and IL-21 (18, 106)], are crucial in regulating the Tfr/Tfh balance. Indeed, the interactions of Tfh and Tfr cells are more complex than discussed above. Recent evidence indicates that Tfh cells can convert into Tfr cells in an IL-2 dependent manner, providing new insights into regulation of Tfr/Tfh balance (107). Moreover, in a breakthrough discovery, Wu et al. (108) found a distinct subset of Tfh cells that could promote Tfr cell differentiation by blocking the Wnt-β-catenin axis in a sclerostin domain-containing protein 1-dependent (SOSTDC-1) manner. Elucidating the cellular and molecular mechanisms underlying the activities of Tfh and Tfr cells in the GC response as well as predominant determinants of the Tfr/Tfh balance will contribute to the regulation of autoantibody production.

### Dysregulated Tfh/Tfr in Autoimmune Diseases

Investigations of the function of Tfh and Tfr cells in regulating antibody production have promoted the discovery of detailed roles for these cells in the pathogenesis of autoimmune diseases. In various established mouse models of autoimmune diseases, both Tfh and Tfr cells are dysregulated. For example, the frequency of Tfr cells in the spleens of BXD2 mice is reduced, whereas Tfh frequency is significantly increased and positively correlated with the frequency of GC B cells (109). It has also been demonstrated that mice deficient in Tfr cells are more prone to developing experimental Sjögren’s syndrome (ESS) than wild-type mice (110). By contrast, the transfer of Tfr cells into BXD2 mice suppressed GC development (18), indicating the crucial role of these cells in immune tolerance.

Studies on Tfh and Tfr cells in human autoimmune diseases are largely restricted to circulating Tfh (cTfh) and Tfr (cTfr) cells, given the difficulty obtaining human secondary lymphoid organs, although the GC response usually occurs in lymphoid organs. cTfh cells can home to LNs and have the superior capacity to provide help for B-cell activation, whereas cTfr cells have a much lower suppressive capacity than LN Tfr cells (98). Notably, these cells are derived from peripheral lymphoid tissues (111) and have comparable Foxp3 expression with tonsil-derived Tfr cells (112). After special activation, cTfr cells can be recruited to GCs to exert suppressive function (98). As a consequence, cTfh and cTfr cells are also closely related to the GC response. It is possible that cTfh cells and cTfr cells vary with alterations in the germinal center, making them indicators of humoral activity and disease severity (111).

Alterations in cTfr cells and cTfh cells occur in a variety of human autoimmune diseases. Patients with autoimmune diseases such as RA (15, 113), SLE (16, 114), myasthenia gravis (MG) (17), primary biliary cholangitis (PBC) (115), and antineutrophil cytoplasmic antibody-associated vasculitis (116) often have an uncontrolled expansion of Tfh cells and decreased Tfr cells in the blood. In MS patients, the functions of cTfr cells are significantly impaired (112) and the cTfr/cTfh ratio is conversely related to IgG production (117). More importantly, the ratio of cTfh/cTfr is inversely correlated with disease activity in many autoimmune diseases (15, 118, 119). Taken together, these findings raise the possibility that impairment of Tfr cells as well as excessive Tfh activity are implicated in the pathogenesis of autoimmunity.

Inconsistent with the above findings, recent studies have described an increase of cTfr cells in SLE (120), RA (121), Sjögren syndrome (111, 122), and AS (123), as well as unchanged frequency of total cTfh cells in muscle-specific kinase MG (MuSK-MG) patients (124). This can be explained by the heterogeneity of patients in different studies. Alternatively, the increase in Tfr cells can also be considered as feedback to enhance the Tfh response in these patients; however, this feedback might be insufficient. In a study of RA, the Tfr/Tfh ratio decreased, despite increases in both cTfh and cTfr cells (118). Another explanation for cTfr increasing in some studies may be that the proportion of activated cTfr cell subsets is reduced, although overall cTfr cells are increased, ultimately leading to an excessive GC response. Consistently, studies of SLE patients have indicated that the active phenotypes of cTfh and cTfh cells are altered and might lead to autoimmunity, whereas overall cTfh and cTfr cells are not significantly different between patients and healthy controls (107). The recent description of Th2- and Th17-like cell subsets of cTfr cells representing ongoing humoral responses in pSS also supports the imbalance of cTfr subsets in autoimmune diseases to some extent (125). Moreover, observations from Li et al. (124) in MuSK-MG patients support a key role of Tfh17 cells in blood, but not total Tfh or activated Tfh cells, in the activation of B cells, which indicated cTfh subpopulation are also imbalanced in autoimmunity.

Taken together, besides the alternations in frequency and function, the imbalance of specific Tfr cell subsets and Tfh cells subsets in circulating also appears to account for the uncontrolled GC response in autoimmune diseases. The functional diversity of Tfh and Tfr cells in different complex immune compartments such as spleens, Peyer’s patches, CLN, joints synovia of RA, and salivary glands of patients with SS are largely unknown. Although difficult, isolation of Tfh and Tfr cells from human tissue will greatly contribute to uncovering the function and underlying mechanism of these cells in autoimmune diseases in the future.

## Regulation of Bregs by Tfh and Tfr Cells

The development and function of Bregs are likely to be regulated by Tfh cells. One of the early lines of evidence from MRL/lpr mice revealed that B10 cells in the spleens were expanded and the percentage of these cells had a strong positive correlation with Tfh percentage (126). In *vitro* data also showed that supernatants from cultured Tfh cells induced IL-10 production by B10 cells, and this impact could be eliminated by neutralization of IL-21 (126). In this regard, the author speculated that Tfh cell-derived cytokine IL-21 drove the differentiation and IL-10 production of B10 cells. Further data showed that this mechanism was related to the activation of phosphorylated -STAT3 by IL-21 (126). A similar study also revealed that cTfh cell from SLE patient contributed to the expansion of CD19^+^CD5^+^CD1d^hi^Bregs (34). This finding proposed that the induction of CD19^+^CD5^+^CD1d^hi^Bregs by Tfh-derived IL-21 was regulatory feedback. Indeed, the importance of IL-21 in promoting CD19^+^CD5^+^CD1d^hi^Bregs generation has already been identified in mice with MS (62). Moreover, Tfh cells as the predominant origin of IL-21 in GC have also been reported (127). This study on SLE linked IL-21 to Tfh-mediated regulation of B cells, providing important evidence that Tfh cells can induce CD19^+^CD5^+^CD1d^hi^Bregs, possibly through IL-21 in autoimmune diseases. Most recently, it was found that CD25+Foxp3+ Treg-like Tfh cells, a specific subset of Tfh cells with the capacity to produce IL-21 during chronic hepatitis B virus (HBV) infection, not only promote the differentiation of B cells into IL-10^+^CD10^-^CD27^-^CD19^+^ Bregs but also enhance the suppressive function of Breg (128). It is conceivable that excessive Tfh cell responses evoke reactive differentiation of Bregs both in autoimmunity and infection. The upregulation of Bregs by Tfh cells can be considered as a potential regulatory feedback mechanism to inhibit proinflammation response. We speculate the function of these Tfh cell-induced Bregs are likely impaired, which eventually led to autoimmunity.

Studies regarding the regulatory properties of Tfr cells on Bregs in autoimmune diseases are rare. However, in acute respiratory distress syndrome, expanded Tfr cells reportedly significantly increase IL-10+Breg cell frequency significantly *in vitro*, extending the understanding of Tfr cell function in immune suppression (129). A recent study in atherosclerosis also indicated that Tfr cells are able to promote B220^+^CD43^-^CD1d^hi^CD5^+^Bregs generation both *in vivo* and *in vitro*. In this study, transfer of Tfr cells into mice with atherosclerosis triggered a significant expansion of B220^+^CD43^-^CD1d^hi^ghCD5^+^Bregs (130). *In vitro* studies implicating the Tfr cell-mediated increase of B220^+^CD43^-^CD1d^hi^ghCD5^+^Bregs requires direct cellular contact and was in proportion to the number of Tfr cells (130). Besides, this effect relies on the presence of Tfh cells (130). It is possible that excessive Tfh activity initiate a series of Tfr-mediated anti-inflammatory responses, the expansion of B220^+^CD43^-^CD1d^hi^ghCD5^+^Bregs Bregs is one of them. Yet Breg expansion in this study may also due to the direct induction by Tfh cells. To date, similar findings in autoimmune diseases have been absent. Further studies are needed to determine how Tfr cell functions to regulate Bregs and whether this impaired regulation correlates with the development of autoimmune diseases.

## Novel Insights Into BREG-Mediated Regulation of Tfh and Tfr in Autoantibody Production

### Bregs Regulate the Differentiation and Function of Tfh and Tfr Cells

As a potent regulator, Bregs have extensive impacts on various immune cells in complex immune microenvironment (19). The interactions between Bregs and their target cells is a hot topic in immune regulation. Recent evidence suggests the emerging role of several Breg cell types in the regulation of Tfh cell differentiation and function. Tfh and B cells co-culture experiment has shown that the addition of CD19^hi^IgD^+^CD38^hi^CD24^hi^CD40^hi^PD-L1^+^IL-21R^+^ human Bregs in the system significantly inhibit Tfh cell maturation, while Tfr cells proportion was increased significantly (35). These Bregs also preclude mature Tfh cells-mediated plasma cell survival and IgM, IgG, and IgA production, suggesting an important role of Breg in inhibiting Tfh cell function (35). Consistently, in a variety of diseases, the number and function of Bregs are decreased, and they are also strongly correlated with the frequency and function of Tfh cells. For example, the study conducted on pSS patients have described an inverse relationship between Tfh percentage and IL-10^+^CD19^+^CD24^+^CD38^hi^ Breg percentage in the circulating (32). The authors further confirmed that IL-10-producing ability of these Bregs in pSS patients was significantly impaired than that in healthy controls, and these Bregs could not effectively inhibit autologous Tfh cell expansion (32). Studies performed in mouse models of EAE (33) and atherosclerosis (36) have further confirmed that PD-L1^+^ Bregs and MZB cells could inhibit the development of Tfh cells and Tfh cell-mediated proatherogenic response, eventually abrogating the acceleration of diseases. Thus, Bregs regulate Tfh cell response and this regulation are more likely to be impaired during various inflammatory diseases.

The detailed Breg regulatory mechanism of Tfh cells differentiation and function are largely undefined and are suggested to rely on direct Breg-Tfh cell contacts and several soluble factors (35). Particularly, Breg-derived IL-10 may play a central role in Tfh cells regulation. Lin et al. (32) found that CD19^+^CD1d^hi^CD5^+^Breg from mice suppressed Tfh cells differentiation in an IL-10-dependent manner *in vitro*. Adoptive transfer of IL-10−/− B cells into ESS mice led to higher GC Tfh and plasma cell accumulation than control mice transferred with WT B cells. They also detected high expression of IL-10 receptor on Tfh cells in mice and man, suggesting the potential role of IL-10 in the regulation of Tfh (32). Importantly, IL-10 downregulated the expression of transcription factor achaete-scute homologue 2 (Ascl2) in Tfh cells (32). Ascl2 is a key regulator during Tfh cells development (131). Downregulation of Ascl2 is thought to inhibit CXCR5 expression, which results in impaired Tfh differentiation and function as CXCR5 is constitutively expressed in Tfh cells and is critical for the maturation and activity of these cells (86, 132, 133). Notably, p-STAT5 inhibition can eliminate IL-10-mediated suppression on Tfh cells (32). These findings suggest the important role of the IL-10-pSTAT5-Ascl2-CXCR5 axis in Tfh cell differentiation and function. Moreover, PD-1/PD-L1 signaling is also involved in Breg-mediated inhibition of Tfh cells. Splenic B cells show the enhanced capacity to promote functional transcription factor Bcl-6 expression in Tfh cells after blocking PD-L1 on B cells (134). PD-L1- abrogated MZB cells are unable to limit the proatherogenic Tfh response and caused severe atherosclerosis (36). The author further found that MZB cells suppress Tfh cell motility in a PD-L1-mediated manner, which may be associated with the impaired capacity of Tfh cells to provide help for B cells (36).This is in agreement with a previous study which indicated that PD-L1^hi^ Bregs require the high expression of PD-L1 to repress Tfh expansion in EAE mouse models (33). Notably, it is possible that Tfr cells participate in Breg-mediated regulation of Tfh cells, given that the differentiation and function of Tfh cells are restricted by Tfr cells. However, after the transfer of PD-L1hi Breg, the percentage and number of Tfh cells in mice were markedly decreased without an increase in Tfr cells, suggesting that this Breg cell type might act directly on Tfh cells rather than *via* Tfr cells (33). Additionally, MZB cells inhibit the accumulation of Tfh cells and GC B cells in the spleen to achieve atheroprotective effects, and the activating transcription factor 3 plays a central role in this effect (36). The expression of transcription factor CTLA-4 is downregulated in Tfh cells when MZB cells are deficient, making Tfh cells susceptible to being activated, suggesting the important role of MZB cells in suppressing the activation of Tfh cells (36). Notably, CTLA-4 expressed at a high level in Tfr cells is critically linked to the suppressive capacity of these cells (135). Considering the role of MZB cells in the maintenance of CTLA-4 in Tfh cells, it will be interesting to determine whether MZB cells affect the function of Tfr cells.

Notably, current studies indicate that Tfh cells contain three subsets with different expression of chemokine receptors, including Tfh1, Tfh2, and Tfh17 (136). Thus, studies on the impact of various Bregs on Tfh cells should evaluate the level of these cellular subsets. In patients with idiopathic pulmonary fibrosis, a decrease in circulating CD3^-^CD19^+^CD24^hi^CD27^+^Bregs has been observed, accompanied by the altered profile of Tfh-cell subsets, in which the proportion of Tfh2 cells and PD-1^+^ICOS^+^-activated Tfh cells are elevated while Tfh17 cells are decreased (137). Similarly, Tfh2 skewing and CD19^+^CD24^hi^CD27^+^Bregs decrease also occurs in the peripheral blood of patients with allergic rhinitis (AR) and AR combined with Asthma (138, 139). The %Tfh2 cells per %CD19^+^CD24^hi^CD27^+^Bregs had a positive correlation with the levels of biomarkers of allergic airway inflammation, making it an exaggerating factor during AR progression to AR with asthma (138). Furthermore, the cTfh2/CD19^+^CD24^hi^CD27^+^Bregs ratio correlates with plasma levels of CXCL13 in asthma (139). Studies in autoimmune diseases previously showed that CXCL13 indicated the disease activity (140–142). Thus, the cTfh2/CD19^+^CD24^hi^CD27^+^cBreg ratio might represent a useful biomarker for diagnosis, severity, and treatment efficacy of autoimmune diseases. Taken together, these data raise a question about the impact of Bregs on different subsets of Tfh cells, especially Tfh17 and Tfh2, two subsets of Tfh cells reported to be correlated positively with antibody production in several autoimmune diseases such as myositis (136), vasculitis (143) and Sjögren’s syndrome (144). It is possible that the functional and/or numerical deficit of Bregs contributes to the polarization of Tfh2 and underlies the development of autoimmune diseases. However, after asthma treatment, the symptoms and cTfh2 skewing were improved, while the percentage of CD19^+^CD24^hi^CD27^+^Bregs was not significantly different (139). This finding indicates other mechanisms, independent of Bregs function, operate in the regulation of Tfh cell subsets, which require more in-depth studies.

Although Tfr cells have similar phenotypes to Tfh cells, studies regarding the role of Bregs in regulating Tfr cell generation are rare. IL-10 deficiency in B cells caused decreased Tfr cells in the B cell follicles (26). One *in vitro* assay cultured Tfh and CD19^hi^IgD^+^CD38^hi^CD24^hi^CD40^hi^PD-L1^+^IL-21R^+^ Bregs sorted from humans showed that the proportion of Foxp3+ cells was increased, suggesting this type of Bregs can induce Tfr cells (35). The increased level of TGF-β in the cocultures and diminished frequencies of Foxp3+ T cells after anti-TGF-β blocking antibodies indicated the requirement for TGF-β in the induction of Tfr cells by these Bregs (35). Remarkably, the effects of several Bregs types on Tregs, a subset of T cells known to differentiate into Tfr cells (8, 10), have been extensively described. CD19^+^CD24^hi^CD38^hi^ Bregs (28) and CD19^+^CD25^hi^ Bregs (71) from human as well as IL-10^+^CD1d^hi^CD5^+^ Bregs (145) and B220^+^CD23^+^T2-MZP B cells (37) from mice all have the capacity to induce Tregs through IL-10 production. Thus, it is conceivable that Bregs promote the development of Tfr cells *via* inducing Treg cells. Studies have also shown that IL-10 deficiency in B cells leads to impaired Tfr cells differentiation and prevent tolerance to the allogeneic cardiac allograft, indicating an important role for IL-10 in B cells-mediated regulation of Tfr cells (26). Notably, IL-10 production is not unique to Bregs. Tfh and Treg cells reportedly produce IL-10 (146, 147). The possibility that IL-10 produced by these cells is also involved in the Tfr cells regulation cannot be excluded.

Interestingly, the ability of different subsets of Bregs to regulate Tfr cells appears to be distinct, due to their different functional markers. For example, human IL-10-producing CD19^hi^IgD^+^CD38^hi^CD24^hi^CD40^hi^PD-L1^+^IL-21R^+^ Bregs expand Tfr cells (35), while CD3^-^CD19^+^PD-L1^hi^ Bregs in mice exhibit a negative effect on the generation of Tfr cells (33). Considering that Tfr cells express large amounts of PD-1 which inhibit Tfr cell development (135), this difference might be attributed to the interactions between Tfr cells and PD-L1^hi^ Bregs through PD-1/PD-L1. Certainly, species differences might also account for this difference, which needs further confirmation.

**Figure 2:**
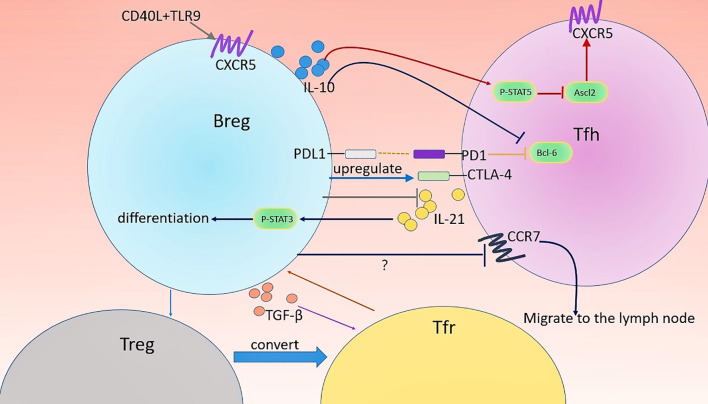
Regulation of Tfh and Tfr cells by Bregs. Several types of Breg are involved in the regulation of the differentiation, function, and distribution of Tfh and Tfr cells. Firstly, CD40 and TLR9 might favor the migration of CD19^hi^IgD^+^CD38^hi^CD24^hi^CD40^hi^PD-L1^+^IL-21R^+^ human Bregs into GC by promoting CXCR5 expression. CD19^+^CD1d^hi^CD5^+^Bregs-derived IL-10 downregulates the expression of CXCR5 in Tfh cells by inhibiting Ascl2, the positive regulator of CXCR5, leading to impaired maturation and Il-21 production of Tfh cell. Moreover, MZB cells play a crucial role in the maintenance of CTLA-4 in Tfh cells. The location of Tfh cells can affect their function. IL-10^+^ MZP B cells direct this migration of Tfh cells out of follicles possibly through altering the expression of CCR7 (crucial chemokine receptors required for the localization of T cells in the LN) in Tfh cells. In turn, Tfh cell activates p-STAT3 in an IL-21-depadent manner therethrough driving the differentiation of CD19^+^CD5^+^CD1d^hi^Bregs. CD19^hi^IgD^+^CD38^hi^CD24^hi^CD40^hi^PD-L1^+^IL-21R^+^ human Bregs can promote Tfr cell differentiation and TGF-β play an important role in this process. Possibly, these Bregs induce Treg through IL-10 production and thereby increase Treg conversion into Tfr cells.

Collectively, all these data support the fact that multiple Bregs regulate the function and differentiation of Tfh cells and Tfr cells through complex mechanisms (See **Figure 2**). Possibly, Bregs balance Tfh and Tfr to ensure central tolerance and prevent autoantibodies production, and dysregulation of Tfh/Tfr cells in autoimmune diseases may due to defective Bregs regulation. Notably, different subsets of Breg and/or different contexts of diseases may cause distinct regulatory effects on Tfh and Tfr cells. The detailed mechanisms of underlying this regulation need further study.

### Bregs Appear to Be Critical to the Distribution of Tfh and Tfr Cells

In addition to the function and frequency, the spatial distribution of Tfr cells in the immune environment also appears to be critical to the induction of immune tolerance (99). In particular, Tfr cells at the T-B border and within the follicle, but not in the GC, have the most efficient ability to mediate immune suppression (99). Studies using tolerogen-treated mice have shown that selective deletion of IL-10 in B cells results in reduced localization of both Tfr and Tfh cells in B cell follicles, and, in contrast, increased Th17 cells in the GCs of lymph node (LN) and the spleen (26). This distribution of Tfr and Tfh cells can be restored by adoptive transfer of IL-10^+^ MZP B cells (26). This study showed that the distribution of Tfh, Tfr, and Th17 cells in secondary lymphoid organs was at least partly controlled by Bregs and this function might be mediated by MZP B cells-derived IL-10, indicating a novel regulatory mechanism for T cell distribution in the GCs. However, it remains largely unknown whether a similar migratory behavior is performed in human. Moreover, the question of what detailed mechanisms are involved in this migration remains. CCR7 are crucial chemokine receptors directing T cells residing in the T zone and down-regulation of CCR7 is indispensable for the follicular positioning of Tfh cells (133). It is possible that MZP B cells downregulate the expression of CCR7 so that Tfh cells can migrate into B cell follicles. However, B cell depletion leads to reduced CCR7 in Tfh cells accompanied by decreased migration of Tfh cells into B cell follicles (26). Thus, the function of MZP B cells in supporting the migration of Tfh cells through CCR7 needs further confirmation. Notably, cTfh cells with low CCR7 expression are increased and have been described as an indicator in autoimmune diseases including AIH (148) and pSS (125), as well as chronic HBV infection (149). CCR7^int^Tfh cells in the blood are closest to tonsillar Tfh lineage cells and exhibit a more potent capacity to induce memory B cells to differentiate into antibody-producing cells than CCR7^high^Tfh cells (150). Therefore, downregulating CCR7 may be important for Tfh cells in promoting antibody production. The effective regulation of GC response may be achieved by altering Tfh cell distribution in the future.

Indeed, the migration of Tfh and Tfr cells into the GC is a complex mechanism with multiple factors involved. Besides CCR7, other elements are also being elucidated. CXCR5 is one of the important molecules guiding the residence in GCs (10, 86, 133). Multiple types of stromal cells in secondary lymphoid organs, including FDCs and fibroblastic reticular cells, which expresses directing signals and provide an important platform for cellular interactions, are also critically determine the GC localization of Tfh and Tfr cells (151). Importantly, activated FDCs express amounts of CXCL13, the ligand for CXCR5, inducing the migration of CXCR5-expressing T and B cell into follicles (132, 152). Whether Bregs change the expression of CXCRL13 in FDCs to mediate the migration of Tfh and Tfr cells warrants further study (26). Notably, positive regulator sphingosine-1-phosphate receptor 2 (153) and negative regulator ephrin-B1 (154) are also involved in the retention of Tfh cells in the GC microenvironment. Thus, the mechanism by which Bregs to regulate the distribution of Tfr and Tfh cells may not be confined to CXCR5. The presence of CXCR5-deficient Tfr cells in the GC also suggest that CXCR5-independent mechanisms operate in their location in the GC (155).

Whether abnormal distribution impacts on the effector functions of Tfh cells and Tfr cells remains unclear. A recent study conducted in fresh human adenoids showed that Tfh cells in distinct locations differed in their motility and functions (156). In outer zones of GC, Tfr cells are fast-migrating and exhibit brief cellular interaction while the majority of Tfr cells in the central GC zone are static with long-lasting capacity to interact with each other (156). Thus, it is possible that the altered location of Tfh and Tfr cells in the GC of secondary lymphoid organs may lead to their changes in function, in parallel. Notably, studies conducted in sarcoidosis have indicated that cTfh cells can migrate into granulomas to play an inflammatory role (157). Thus, the migration of Tfh cells not only exists in the GC microenvironment in one lymphoid tissue or organ but also occurs between different secondary lymphoid organs. Whether Bregs also play a role in directing this distribution between different lymphoid tissues or organs is unknown. Moreover, the impact of Bregs on the migration of Tfh and Tfr cells in the blood and their counterparts in non-lymphoid tissue such as joint synovia of RA patients and salivary glands of patients with pSS warrant further study.

## Therapeutic Prospects of Bregs-Mediated Tfr/Tfh Regulation

Given the considerable role of autoantibodies in the development of autoimmune diseases, current therapies focus on eliminating autoantibodies and/or blocking their function in a more precise manner. Particularly, targeting key cells involved in autoantibodies production is more popular due to the high efficacy and less adverse effects. In this regard, Tfr and Tfh cells modulation strategies have potent potential, for the contrasting roles of Tfr and Tfh in the regulation of GC response (158). However, therapies targeting Tfr and Tfh cells directly are extremely limited, due to a poor understanding of their development and function.

Strikingly, specific subsets of Bregs hold considerable promise to regulate Tfr/Tfh balance (26, 32, 33, 35, 36). As mentioned above, defective Bregs may lead to aberrant differentiation, function and distribution of Tfr and Tfh cells in diseases. Indeed, Breg number is decreased and the cross-talks between Breg and their target cells are also compromised in multiple autoimmune diseases (27, 159–161). For instance, in SLE, CD19^+^CD24^hi^CD38^hi^ Bregs were unable to restrain IFN-α production by pDCs. pDCs also failed to drive these Bregs differentiation (31). In pSS, CD19+CD24+CD38hi Breg were defective in suppressing the expansion of Tfh cells (32). IL-10^+^CD19^+^CD24^hi^CD38^hi^ Bregs from thyroid associated ophthalmopathy patients also failed to activate IFN-γ+ and IL-17+ T cells (162). Accordingly, therapies of selectively transferring and/or inducing Bregs, such as CD1d^hi^CD5^+^Breg and CD19^+^CD25^+^CD1d^hi^IgM^hi^Breg, have been developed to restore homeostasis and shown some success in mice disease models, which provide evidence for the application of Bregs in the treatment (40, 55, 163, 164). Importantly, in mice models of experimental Sjögren’s syndrome (ESS), adoptive transfer of CD19^+^CD1d^hi^CD5^+^ Bregs effectively suppressed the Tfh cell response, leading to the amelioration of diseases progression (32). It is noteworthy that multiple strategies to induce Breg cell through key molecules, such as anti-CD40 mAb (163), BAFF (63), enteric microbiota (61, 66), and bacteria-derived oligodeoxynucleotides (CpG-ODN) (67), may also indirectly affect the balance of Tfh/Tfr cells; however, studies in this regard are scarce. Furthermore, as discussed above, the immunosuppressive function of Breg is mostly related to IL-10 secretion. IL-10 would be promising to regulate the differentiation, function and migration of Tfr and Tfh cells. However, IL-10 also acts as a B cell growth factor and can promote autoantibody production (165). The application of IL-10 into Tfr and Tfh cells regulation may cause unwanted pro-inflammatory effects. In addition to treatment, Bregs may be an excellent indicator for evaluating the efficacy and prognosis of diseases. A higher ratio of pre-transplant cTfh/IL10^+^CD19^+^CD24^+^CD38^+^ Bregs is reportedly correlated with graft rejection (166). It may be worth to determine whether this ratio contributes to assessing disease activity and correlates with the level of autoantibody in autoimmune diseases.

However, considering the exact mechanisms underlying the interplays of Breg, Tfh cells, and Tfr cells in health and patients are largely unknown, it is still of great challenge to achieve precise regulation of Tfh and Tfr cells through Bregs. For example, selectively induction of immunosuppressive B cells without activating effector B cells *in vivo* is still difficult, although the great plasticity of Bregs makes it possible to induce these cells in the various microenvironment. Meanwhile, it is hard to know how to make an adequate induction of Bregs. Excessive or insufficient induction of Breg may lead to various dysregulation of the immune system. The safety and efficacy also needed to be determined in more animal and clinical trials in the future. Moreover, Bregs have vast plasticity in disease microenvironments and can differentiate into other kinds of B cells subset including effector B cells. The maintenance of Bregs immunosuppressive function *in vivo* after transferring and/or inducing these cells in patients must be taken into consideration. Another limiting factor for Breg immunotherapy is the identification of multiple immunosuppressive Bregs types in humans. Bregs are extremely heterogeneous and not all types of Bregs equally regulate Tfh and Tfr cells.

Taken together, Breg-mediated Tfr/Tfh regulation provides novel insights in limiting the exaggerated autoantibody production without broad immunosuppression in autoimmune diseases.

## Conclusions

As demonstrated in numerous reports within the literature, Tfh/Tfr balance is necessary to maintain proper antibody production, and this balance has strong links with several types of Breg, such as PD-L1^hi^B cells, IL-10^+^CD19^+^CD24^+^CD38^hi^ Bregs, and MZB cells. These Bregs not only regulate the differentiation and function of Tfr and Tfh cells but also appears to affect their distribution in the immune microenvironment. More importantly, Bregs may achieve the fine tune of Tfh and Tfr cells in the level of subpopulations, which contribute to restore the balance of Tfr and Tfh subpopulation in autoimmune diseases. Thus, Breg-based therapy has theoretical feasibility and clinical application prospect to regulate the activity of Tfh and Tfr cells and autoantibody production in autoimmune diseases. However, A number of questions on the development, phenotype, and function of Bregs remain to be answered, which restrain the translation of Bregs into clinical application. A complete understanding of Breg-Tfh cell and Breg-Tfr cell cross-talks in health and autoimmune diseases is needed, which will provide the rationale for designing more effective immunotherapy in autoimmune disorders.

## Author Contributions

TD drafted the manuscript, prepared illustrations, and discussed the content with the other authors. CW conceived the topic and revised the content of the manuscript. RS, RW, HX, YW, and RHS revised the manuscript. CG, and XL also critically revised the content of the manuscript. All authors contributed to the article and approved the submitted version.

## Funding

This work was supported by the National Natural Science Foundation of China (No. 81971543), Natural Science Foundation of China (No. 81471618), and Key Research and Development (R&D) Projects of Shanxi Province (201803D31119).

## Conflict of Interest

The authors declare that the research was conducted in the absence of any commercial or financial relationships that could be construed as a potential conflict of interest.
